# Improvement of Kiteplatin Efficacy by a Benzoato Pt(IV) Prodrug Suitable for Oral Administration

**DOI:** 10.3390/ijms23137081

**Published:** 2022-06-25

**Authors:** Alessandra Barbanente, Valentina Gandin, Cecilia Ceresa, Cristina Marzano, Nicoletta Ditaranto, James D. Hoeschele, Giovanni Natile, Fabio Arnesano, Concetta Pacifico, Francesco P. Intini, Nicola Margiotta

**Affiliations:** 1 Dipartimento di Chimica, Università degli Studi di Bari Aldo Moro, Via E. Orabona 4, 70125 Bari, Italy; alessandra.barbanente@uniba.it (A.B.); nicoletta.ditaranto@uniba.it (N.D.); giovanni.natile@uniba.it (G.N.); fabio.arnesano@uniba.it (F.A.); concetta.pacifico@uniba.it (C.P.); francescopaolo.intini@uniba.it (F.P.I.); 2 Dipartimento di Scienze del Farmaco, Università di Padova, Via Marzolo 5, 35131 Padova, Italy; valentina.gandin@unipd.it (V.G.); cristina.marzano@unipd.it (C.M.); 3 Scuola di Medicina e Chirurgia, Università degli Studi di Milano-Bicocca, Via Cadore 48, 20900 Monza, Italy; cecilia.ceresa@gmail.com; 4 Department of Chemistry, Eastern Michigan University, Ypsilanti, MI 48197, USA; jhoesche@emich.edu

**Keywords:** cisplatin, oxaliplatin, kiteplatin, antitumor drugs, oral administration, colorectal cancer, neurotoxicity

## Abstract

Kiteplatin, [PtCl_2_(*cis*-1,4-DACH)] (DACH = diaminocyclohexane), contains an isomeric form of the oxaliplatin diamine ligand *trans*-1*R*,2*R*-DACH and has been proposed as a valuable drug candidate against cisplatin- and oxaliplatin-resistant tumors, in particular, colorectal cancer. To further improve the activity of kiteplatin, it has been transformed into a Pt(IV) prodrug by the addition of two benzoato groups in the axial positions. The new compound, *cis*,*trans*,*cis*-[PtCl_2_(OBz)_2_(*cis*-1,4-DACH)] (**1**; OBz = benzoate), showed cytotoxic activity at nanomolar concentration against a wide panel of human cancer cell lines. Based on these very promising results, the investigation has been extended to the in vivo activity of compound **1** in a Lewis Lung Carcinoma (LLC) model and its suitability for oral administration. Compound **1** resulted to be remarkably stable in acidic conditions (pH 1.5 to mimic the stomach environment) undergoing a drop of the initial concentration to ~60% of the initial one only after 72 h incubation at 37 °C; thus resulting amenable for oral administration. Interestingly, in a murine model (2·10^6^ LLC cells implanted *i.m.* into the right hind leg of 8-week old male and female C57BL mice), a comparable reduction of tumor mass (~75%) was observed by administering compound **1** by oral gavage and the standard drug cisplatin by intraperitoneal injection, thus indicating that, indeed, there is the possibility of oral administration for this dibenzoato prodrug of kiteplatin. Moreover, since the mechanism of action of Pt(IV) prodrugs involves an initial activation by chemical reduction to cytotoxic Pt(II) species, the reduction of **1** by two bioreductants (ascorbic acid/sodium ascorbate and glutathione) was investigated resulting to be rather slow (not complete after 120 h incubation at 37 °C). Finally, the neurotoxicity of **1** was evaluated using an in vitro assay.

## 1. Introduction

**Figure 1:**
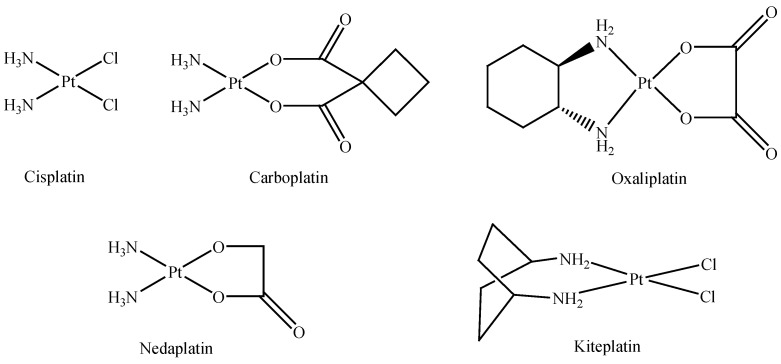
Sketches of clinically used platinum(II) drugs (Cisplatin, Carboplatin, and Oxaliplatin) and of Nedaplatin and Kiteplatin.

Since its discovery, cisplatin has become one of the most widely used anticancer drugs with activity against many tumors [1,2,3,4], such as testicular, ovarian, lung, head and neck, and other types of solid tumors. Its mechanism of action involves cell entry followed by aquation (due to the lower intracellular chloride concentration with respect to that of blood) and binding to purine bases in DNA with the formation of platinum-DNA cross-links that trigger apoptosis [5,6]. Despite the great curative success of cisplatin in testicular cancer, the drug induces side effects [7,8] such as nausea, vomiting, myelosuppresion, immunosuppression, nephrotoxicity, neurotoxicity, and hearing loss. Drug resistance, which can be intrinsic or acquired by tumors, is another major complication arising in platinum-based chemotherapy [9,10]. For this reason, cisplatin analogues (such as carboplatin, oxaliplatin, and nedaplatin, Figure 1) have been developed that are active against cisplatin-resistant tumors and/or have a better therapeutic index (lower toxicity and greater efficacy) [1,11,12,13,14,15]. Kiteplatin, *cis*-1,4-diaminocyclohexane(dichlorido)platinum(II) or [PtCl_2_(*cis*-1,4-DACH)] (DACH = diaminocyclohexane), contains an isomeric form of the oxalilplatin diamine ligand *trans*-1*R*,2*R*-diaminocyclohexane (1*R*,2*R*-DACH) and has been widely investigated as a potential new platinum anticancer drug (Figure 1). In particular, the interest in this compound stems from its activity against both cisplatin (ovarian C13) and oxaliplatin (colon LoVo-OXP)-resistant cancer cell lines [16,17,18,19].

**Figure 2:**
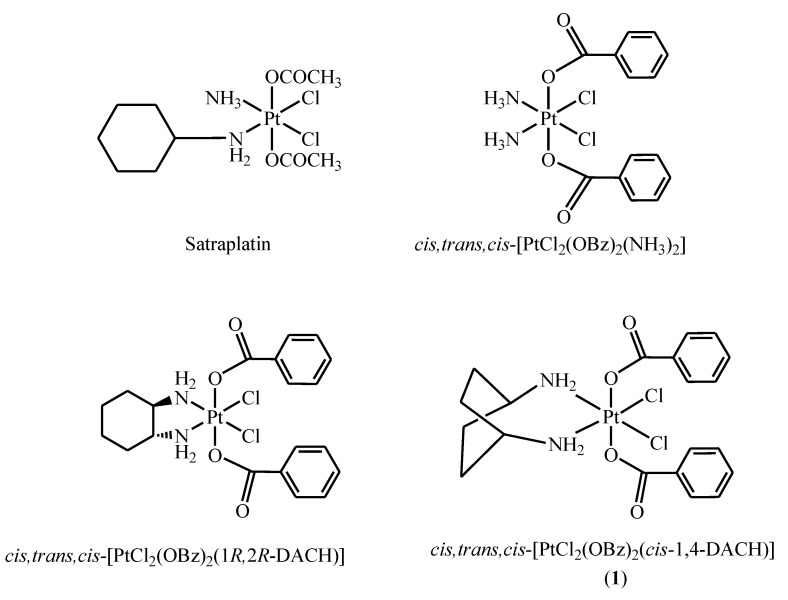
Sketches of Pt(IV) prodrugs (OBz, benzoate; DACH, diaminocyclohexane).

Another strategy to overcome some limitations of conventional Pt(II) anticancer drugs, consists of the synthesis of the corresponding platinum(IV) derivatives to be used as prodrugs [4,20,21,22,23,24]. Pt(IV) complexes are characterized by greater chemical inertness and could therefore be suitable for oral administration [25]. In Pt(IV) complexes, the additional axial ligands also provide the mean to modulate some properties such as reduction potential, lipophilicity, and specificity for given organs or tissues, and in some cases, the ligands themselves can perform, upon release, additional pharmacological effects [4,26,27,28,29,30,31,32,33,34]. Lippard was first to propose the coordination of the axial ligands through carboxylato groups [35]. These Pt(IV) compounds were found to be easily reduced under physiological condition by biological reducing agents (such as glutathione and ascorbic acid) affording the cytotoxic Pt(II) species and the free axial ligands with their pharmacological potential. One prominent example of Pt(IV) prodrugs was satraplatin (*bis*-acetato-ammine-dichlorido-cyclohexylamineplatinum(IV); Figure 2) that reached Phase III clinical trials [36] but has not yet been approved by the United States Food and Drug Administration (FDA) [23].

Based on the observation that the presence of aryl groups can improve the uptake of a drug by conferring greater lipophilicity and better transport across the cell membrane (this is the case of aryl ethers-functionalized glycomers, which proved to be effective growth inhibitors and inducers of apoptosis in human glioblastoma cells [37]), Dyson and Ang introduced for the first time benzoate axial ligands in Pt(IV) derivatives [24,38,39]. The dibenzoate Pt(IV) derivative of cisplatin (*cis*,*trans*,*cis*-[PtCl_2_(OBz)_2_(NH_3_)_2_]; OBz = benzoate; Figure 2) was found to be 30-fold more cytotoxic than cisplatin in the A2780 human ovarian carcinoma cell line but was not able to overcome the cisplatin resistance in the A2780/CisR cell line [24]. Unlike in the case of cisplatin, the dibenzoate Pt(IV) derivative of oxaliplatin, *cis*,*trans*,*cis*-[PtCl_2_(OBz)_2_(1*R*,2*R*-DACH)] (Figure 2), not only showed a better in vitro antiproliferative activity than oxaliplatin toward colorectal cancer cells, but it was also active against the resistant LoVo-OXP cell line [40].

The effect of coordinating two benzoato ligands in axial positions was found to be particularly remarkable in the case of kiteplatin. In an in vitro study, the dibenzoate Pt(IV) prodrugs *cis*,*trans*,*cis*-[PtCl_2_(OBz)_2_(*cis*-1,4-DACH)] (**1**, Figure 2) showed to possess antitumor activity already at nanomolar concentrations, resulting in two orders of magnitude more active than cisplatin toward all tested cell lines [41]. Furthermore, the antitumor activity of **1** was found to be 31 times greater than that of *cis*,*trans*,*cis-*[PtCl_2_(OBz)_2_(NH_3_)_2_] against A549 cells [39] and similar to that of *cis*,*trans*,*cis*-[PtCl_2_(OBz)_2_(1*R*,2*R*-DACH)] against the HCT-15 cell line [40].

Based on the exceptional in vitro antitumor activity of compound **1**, we have decided to extend the investigation to an in vivo model (Lewis Lung Carcinoma) and to seek the possibility of oral administration. Moreover, since the mechanism of action of Pt(IV) prodrugs involves their chemical reduction to cytotoxic platinum(II) species, we have measured the reduction potential of compound **1** by cyclic voltammetry (CV) and investigated by ^1^H-NMR its chemical reduction by two bioreductants (ascorbic acid/sodium ascorbate and glutathione). Finally, since neurotoxicity is one of the major side effects of oxaliplatin, the neurotoxicity of **1** has been evaluated by an in vitro assay.

## 2. Results and Discussion

### 2.1. Stability at Acidic pH

Pt(IV) prodrugs are generally assumed to escape degradation by nucleophiles in the blood and to reach the tumor intact. Once inside the cells, Pt(IV) prodrugs can be activated by reduction, thereby releasing the cytotoxic Pt(II) species and the two axial ligands. However, the stability of Pt(IV) prodrugs in the extracellular environment is not always true; for instance, it was demonstrated that Pt(IV) complexes with haloacetato ligands can undergo rather fast hydrolysis under biologically relevant conditions with release of the two ligands in axial positions and formation of the Pt(IV)-dihydroxido derivatives [31,42].

**Figure 3:**
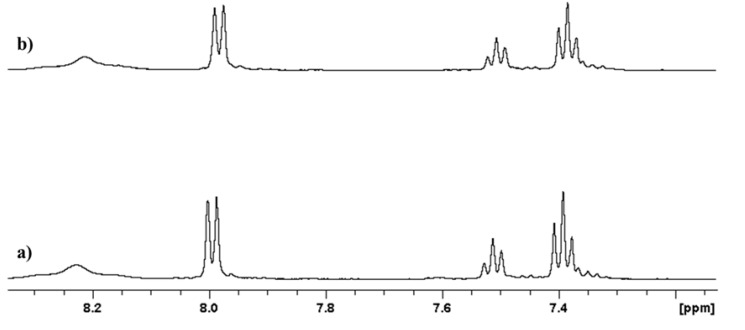
Portion of the ^1^H-NMR (500 MHz) spectra of compound **1** in Acetone-d_6_/D_2_O before (**a**) and soon after (**b**) addition of DCl (pH = 1.5).

In the present case, to gain information about a possible oral administration of the drug, the stability of compound **1** (prepared following the procedure already reported in the literature and fully characterized by spectroscopic and elemental analyses [41]) has been investigated by ^1^H-NMR in Acetone-d_6_/D_2_O solution acidified with DCl (pH = 1.5) as a mimic of the acidic environment of the stomach. The spectra acquired before and soon after the addition of DCl (Figure 3) showed, in the aromatic region, only the peaks of coordinated benzoate ligands located at 8.00, 7.39, and 7.51 ppm, indicating that compound **1** does not undergo rapid decomposition at strongly acidic conditions and in the presence of excess chloride ion.

**Scheme 1:**
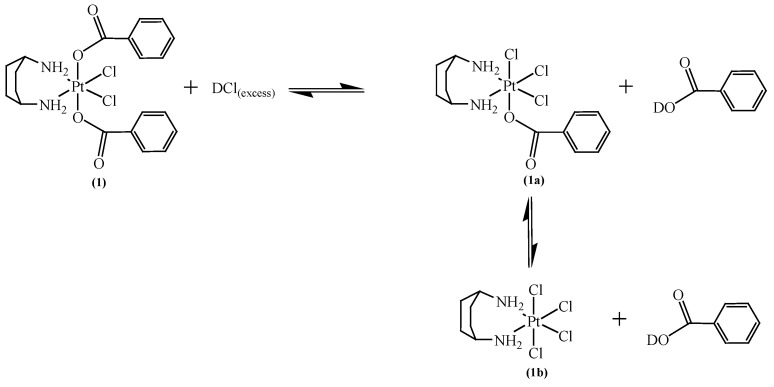
Benzoate substitution by chloride in a compound **1** solution acidified with DCl (pH = 1.5).

The sample was then maintained at 37 °C and NMR spectra recorded at different times in order to investigate the occurrence of substitution reactions at the expense of the benzoato ligands by chloride ions. As shown in Scheme 1, by two consecutive substitution reactions, complex **1** can be converted first into an asymmetric benzoato/chlorido species (**1a**) and then into the tetrachlorido species **1b**.

**Figure 4:**
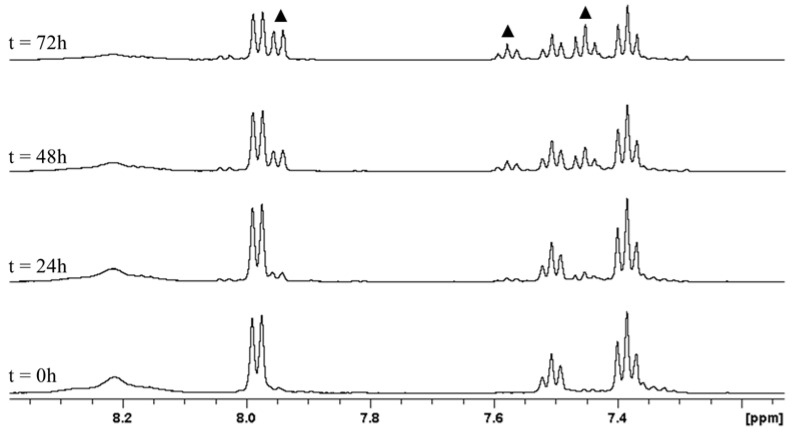
Portion of the ^1^H-NMR (500 MHz) spectra of complex **1** in Acetone-d_6_ acidified with DCl (pH = 1.5), acquired at different time intervals. Free benzoate peaks are marked with black triangles.

The ^1^H-NMR spectra recorded after 24, 48, and 72 h showed a new set of peaks (marked with black triangles in Figure 4) that increased in intensity over time and were assigned to free benzoate. After 72 h, the concentration of complex **1** dropped to ca. 60% of the initial concentration (as calculated by integration of the NMR signals). This experiment clearly shows that compound **1** is sufficiently stable in a strongly acid environment to predict the applicability of oral administration.

### 2.2. Electrochemical Characterization

**Figure 5:**
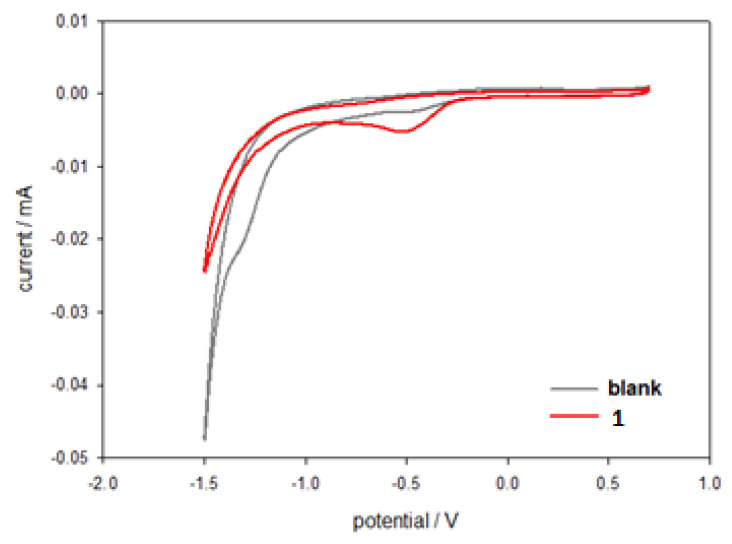
Cyclic voltammogram of **1** recorded with a glassy carbon electrode in an aqueous solution (5 × 10^−4^ M phosphate buffer, pH = 7.4). Scan rate 20 mVs^−1^.

A parameter that gives information on the possibility to obtain intracellular reduction of a Pt(IV) prodrug is the cathodic reduction potential (E_p,c_). This parameter can be obtained by cyclic voltammetry (CV) experiments. Thus, the electrochemical characterization of compound **1** was performed via CV by dissolving the compound at a concentration of 5 × 10^−4^ M in 10 mL phosphate buffer (pH = 7.4, I = 0.1) and NaCl 0.5 M. The cyclic voltammogram (Figure 5) shows an irreversible 2e^−^ reduction of the Pt(IV) species at E_p,c_ = −0.467 V (E_2_C mechanism of bimolecular elimination reaction). The irreversibility is due to the detachment of the two axial ligands upon reduction of the octahedral Pt(IV) complex to the square–planar Pt(II) species.

As already found for Pt(IV) complexes having the same equatorial ligands but differing for the axial ligands [29,30,43], the dibenzoato Pt(IV) derivative of kiteplatin has a redox potential that is in between that of the tetrachlorido species *cis*-[PtCl_4_(*cis*-1,4-DACH)] (−0.055 V, easily reducible) and that of the dihydroxido species *cis,trans,cis*-[PtCl_2_(OH)_2_(*cis*-1,4-DACH)] (−0.967 V, hardly reducible). This could allow selective bioreduction of compound **1** within tumor cells and not in the blood stream.

### 2.3. Characterization of the Surface Reduction

**Figure 6:**
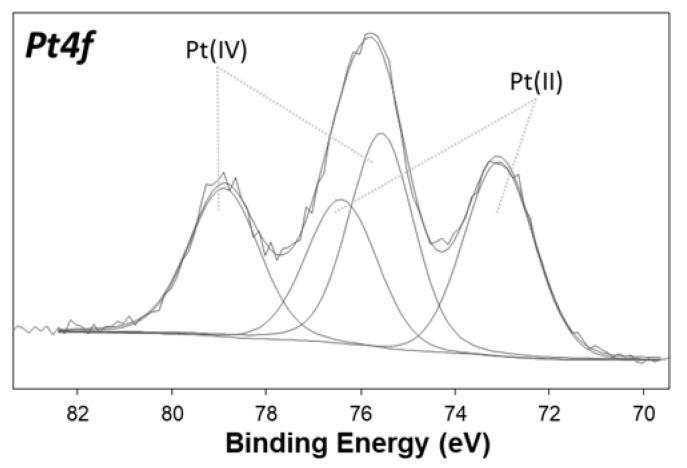
Pt4f XP spectral region of complex **1** along with the curve fitting procedure. Resulting Pt(II) and Pt(IV) signals are indicated.

Compound **1** was also characterized by X-ray photoelectron spectroscopy (XPS) in order to obtain information on the surface chemical composition and on the platinum speciation. Despite the surface sensitivity of the technique (topmost 5–8 nm), the analysis on homogeneous samples provides useful information on the chemical environment of all detected elements. Figure 6 shows the high resolution Pt4f XP spectrum of complex **1**, where the curve-fitting procedure was applied to discriminate all the peak components. Two doublets were found: one is assigned to Pt(II) (Binding Energy, BE Pt4f_7/2_ = 73.0 ± 0.2 eV and BE Pt4f_5/2_ = 76.3 ± 0.2 eV), while the second doublet is assigned to Pt(IV) (BE Pt4f_7/2_ = 75.4 ± 0.2 eV and BE Pt4f_5/2_ = 78.7 ± 0.2 eV). The XPS spectrum clearly shows that some reduction of Pt(IV) to Pt(II) occurred on the sample surface (relative abundance Pt(IV)/Pt(II) = 60:40). Since NMR spectroscopy and ESI-MS, along with elemental analysis, did not show detectable levels of Pt(II) products, we assume that the presence of appreciable amounts of Pt(II) species on the surface is a peculiar characteristic of the XPS sample, whose degradation could be enhanced by X-ray irradiation. The same phenomenon had already been observed for similar Pt(IV) complexes [34].

### 2.4. Reduction of Complex **1** by Bioreductants

**Scheme 2:**
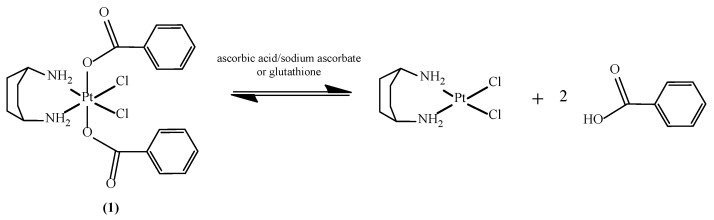
Reduction of complex **1** by the most important intracellular bioreductants with formation of kiteplatin and benzoic acid.

Although numerous reducing agents are present both in the blood and within cells, it is generally recognized that ascorbic acid (vitamin C) and glutathione (GSH) are the major intracellular agents responsible for the activation of Pt(IV) prodrugs into the corresponding bioactive Pt(II) species (Scheme 2).

#### 2.4.1. Reduction of cis,trans,cis-[PtCl_2_(OBz)_2_(cis-1,4-DACH)] by Ascorbic Acid/Sodium Ascorbate

Ascorbic acid is a 2e^−^ reducing agent present in the blood in a much lower concentration (50–150 μM) than the concentration in cells (~1 mM). The reaction between **1** and ascorbic acid was performed in an NMR tube and monitored by ^1^H-NMR. Compound **1** was first dissolved in Acetone-d_6_ while the reducing agent was dissolved in D_2_O. Then, the two solutions were mixed to yield a final solution, which was 550:100 (*v*/*v*) Acetone-d_6_/D_2_O.

**Figure 7:**
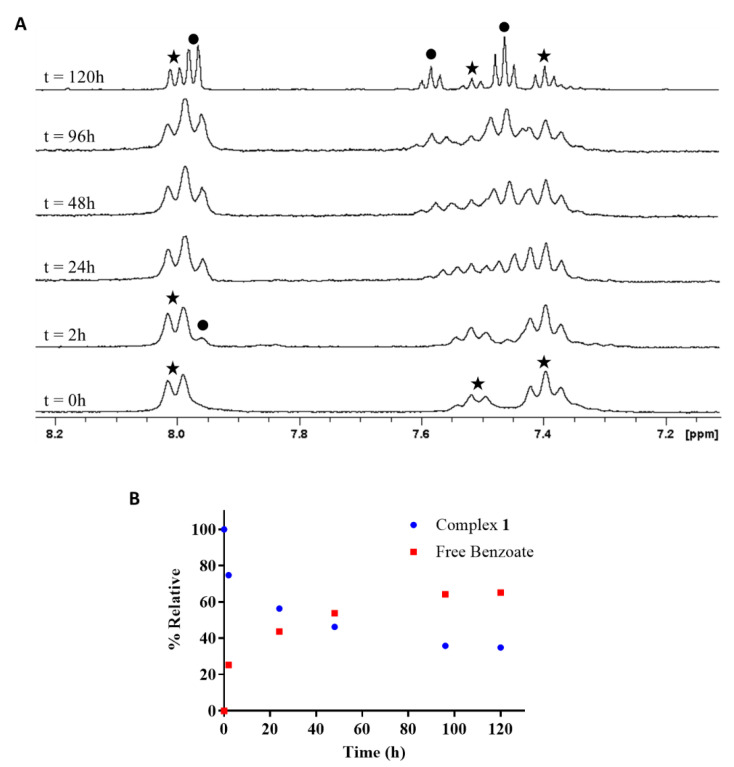
(**A**) Portion of the ^1^H-NMR spectra (300 MHz) of complex **1** treated with an excess (1:6 molar ratio) of a 1:1 mixture of ascorbic acid/sodium ascorbate in Acetone-d_6_/D_2_O (550:100, *v*/*v*) at different time intervals at 37 °C. The spectrum at 120 h was recorded on a 500 MHz instrument. The stars indicate the peaks of the benzoato ligands in starting complex **1**, while the black dots indicate the peaks of free benzoate released after reduction of **1** by ascorbic acid. (**B**) Relative percentages of **1** and free benzoate, obtained by integration of ^1^H NMR deconvoluted signals (8.20–7.80 ppm), plotted as a function of time.

Selected regions of the ^1^H-NMR spectra, recorded at different time intervals, are reported in Figure 7A. The spectrum taken soon after mixing of the reactants (ratio **1**:ascorbic acid = 1:6) shows only peaks at 7.37, 7.49, and 8.00 ppm, which are characteristic of the axial benzoato ligands in compound **1** (marked with stars in Figure 7A). After 2 h at 37 °C, a new signal appears at 7.95 ppm, which belongs to free benzoate (marked with a black dot in Figure 7A). After 24 h at 37 °C, the set of signals belonging to free benzoate gains in intensity. The sample was monitored for 5 days, and the last spectrum (120 h incubation time), recorded on a 500 MHz NMR instrument, which allows a better resolution, clearly shows the peaks of coordinated benzoato in complex **1** (marked with stars) and those belonging to free benzoate (marked with black dots) in a 1:2 ratio. By plotting the relative percentage of complex **1** vs. time (Figure 7B), obtained after deconvolution of NMR signals in the region 8.20–7.80 ppm, it appears that the reduction reaction is not complete and reaches an equilibrium after ca. 100 h.

#### 2.4.2. Reduction of cis,trans,cis-[PtCl_2_(OBz)_2_(cis-1,4-DACH)] by Glutathione

Glutathione (GSH) is a 1e^−^ reducing agent present in cells at a concentration of ~2 mM, approximately twice that as present in the blood stream (~0.9 mM). When GSH acts as a reducing agent, it generates oxidized glutathione (GSSG), a compound that is also capable of reacting with Pt(II) compounds [44,45,46].

**Figure 8:**
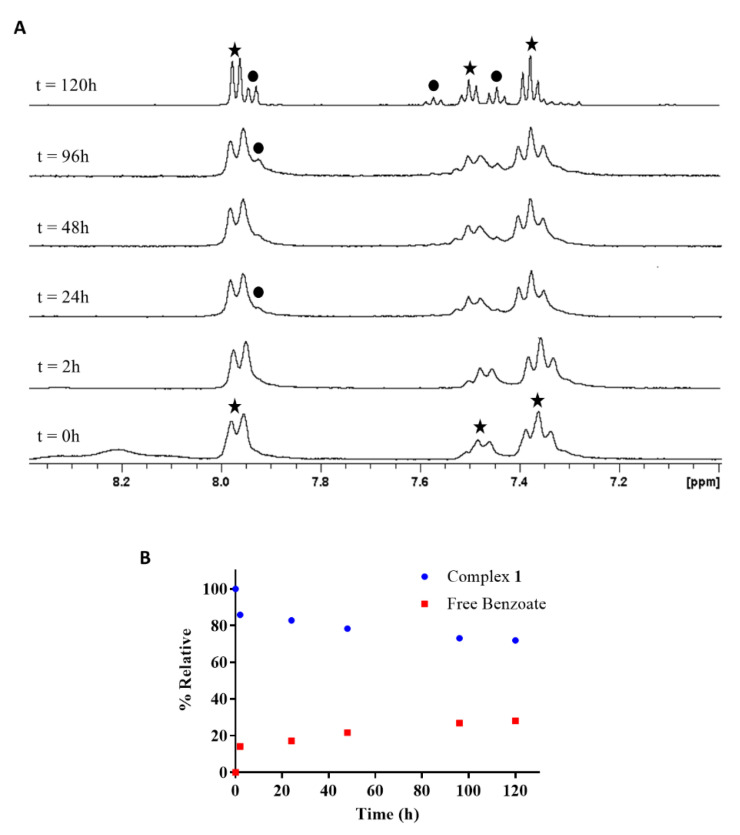
(**A**) Portion of the ^1^H-NMR spectra (300 MHz) of compound **1** treated with an excess of glutathione (1:6 molar ratio) in Acetone-d_6_/D_2_O (550:100, *v*/*v*) at 37 °C, taken at different time intervals. The spectrum at 120 h was recorded on a 500 MHz instrument. The stars indicate the peaks of starting complex **1**, while the black dots indicate the peaks of free benzoate. (**B**) Relative percentages of **1** and free benzoate, obtained by integration of ^1^H NMR deconvoluted signals (8.10–7.80 ppm), plotted as a function of time.

In addition, in the case of the reaction with GSH, we used a mixture of Acetone-d_6_ and D_2_O to achieve dissolution of both reagents. The ^1^H-NMR spectra, recorded at different time intervals, are reported in Figure 8A. The spectrum taken soon after mixing of the reactants shows only peaks at 7.37, 7.49 and 8.00 ppm, which are characteristic of coordinated benzoato ligands in complex **1** (marked with stars in Figure 8A). A tiny signal at 7.95 ppm, belonging to free benzoate (marked with a black dot in Figure 8A), was observed only after 24 h at 37 °C. In addition, in this case, the sample was monitored for 5 days, and the final spectrum (120 h), recorded on a 500 MHz NMR instrument to allow for better resolution, clearly showed the signals belonging to free benzoate, which were markedly smaller than those belonging to the starting complex **1** (~1:2.5 ratio). These results are in agreement with previous kinetic studies highlighting that the reduction of Pt(IV) by GSH is much less efficient than that by a 2e^−^ reductant such as ascorbic acid [47,48]. Moreover, as observed in the reduction reaction by ascorbate, the reduction by glutathione is not complete, and an equilibrium is reached after ca. 100 h (Figure 8B).

### 2.5. In Vivo Antitumor Activity

Based on previous studies indicating that compound **1** was already active at nanomolar concentrations, resulting in two orders of magnitude more effective than cisplatin against all cancer cell lines tested [41], in this study, the in vivo antitumor activity of **1** was tested in the murine model of Lewis lung carcinoma (LLC). Moreover, since compound **1** was found to be stable at acidic pH and excess chloride concentration, it was chosen to administer compound **1** orally to mice. The inhibition of tumor growth induced by **1** was compared to that promoted by the reference metallodrug cisplatin, which instead was administered intraperitoneally. Seven days after tumor inoculation, tumor-bearing mice were randomized into vehicle control and treatment groups (five mice per group). Control mice received the vehicle (0.5% DMSO (*v*/*v*) and 99.5% of saline solution (*v*/*v*)), whereas treated groups received daily doses of **1** (5 mg kg^−1^ in vehicle solution) orally by gavage or cisplatin (1.5 mg kg^−1^ in saline solution) by intraperitoneal injection. The drug treatment regimen in mice may result in accelerated weight loss. In our study, tumor growth was determined as the difference in weight of the tumor-bearing leg and the healthy leg within the same animal; therefore, the weight loss equally affects both legs. The dosage of **1** to be employed for in vivo efficacy studies was selected based on previous experiments assessing the maximum tolerated doses (MTD), which could be injected without undue toxicity in nontumor-bearing C57BL mice [49] and which represent 1/3 of the calculated MTD. Cisplatin treatment schedule was selected according to standard protocols designed to optimize its efficacy and to minimize the occurrence of adverse events. Tumor growth was estimated at day 15, and the results are reported in Table 1. Oral administration of **1** induced 72.5% reduction of the tumor mass compared to that of the control group. The result is comparable to that observed with cisplatin (77.5% reduction), particularly if one takes into account that compound **1** was administered orally while cisplatin was administered intraperitoneally.

### 2.6. Neurotoxicity Studies

**Figure 9:**
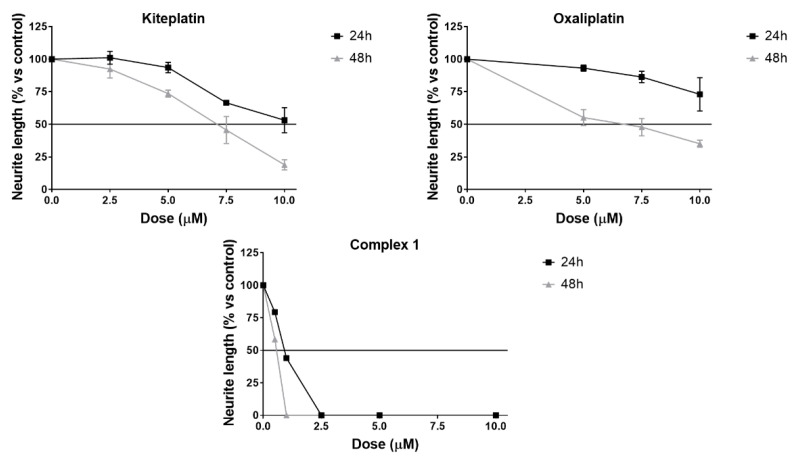
Toxicity in DRG explants. DRG explants from 15-day embryonic Sprague-Dawley rats were treated for 2 h with NGF and subsequently exposed to kiteplatin, oxaliplatin and complex **1** for 24 h or 48 h. Phase-contrast micrographs were taken, and the length of the longest neurite in each DRG was measured using a standard calibration grating photographed at the same magnification.

Peripheral neuropathy is one of the most common dose-limiting adverse effects of cisplatin and oxaliplatin (OXP) and is a major cause of therapy discontinuation [50,51]. OXP is responsible for more than 70% rate of symptomatic neurotoxicity. To investigate the potential neurotoxic effect of compound **1**, we employed a well-established in vitro model based on organotypic cultures of dorsal root ganglia (DRG) from 15-day-old rat embryos. Neurite elongation, under the effect of nerve grown factor (NGF), was evaluated in DRG explants. This model has been widely used to investigate the neurotoxic effect of several anticancer drugs and has given reliable results [52,53,54,55]. For comparison purposes, the effects induced by the reference drugs OXP and kiteplatin were also investigated. A compound is defined as neurotoxic when the mean neurite elongation is reduced by 50% or more after drug exposure vs. control. As expected, after 48 h exposure to OXP, a significant reduced neurite elongation, in a dose-dependent manner, was observed. In particular, OXP at 7.5 µM concentration reduced the neurite length by 50% and this effect is comparable to that observed with kiteplatin (Figure 9). The neurotoxicity of compound **1** was also tested at µM concentrations, notwithstanding its in vitro cytotoxic activity already manifested at nanomolar concentrations. Compound **1** showed a neurotoxic effect at a concentration of ca. 1.0 µM after 24 h of incubation (44% of neurite length compared to the control) and at a concentration slightly higher than 0.5 µM after 48 h of incubation (58% of neurite length compared to the control).

## 3. Materials and Methods

### 3.1. Starting Materials and Instrumental Details

Commercial reagent grade chemicals and solvents were used as received without further purification. ^1^H-NMR spectra were recorded on a Bruker Avance 300 Ultrashield spectrometer (Billerica, MA, USA) and on an Agilent 500/54 Premium Shielded spectrometer (Santa Clara, CA, USA). ^1^H chemical shifts were referenced using the internal residual peak of the solvent (D_2_O: 4.80 ppm; Acetone-d_6_: 2.05 ppm). Electrospray ionization mass spectrometry (ESI-MS) was performed with an Agilent 6530 Accurate-Mass Quadruple Time-of-Flight (Q-TOF) system equipped with an electrospray interface. Elemental analyses were performed with an Eurovector EA 3000 CHN instrument (Pavia, Italy). A Crison Micro-pH meter Model 2002 (Alella, Barcelona), equipped with Crison microcombination electrodes (5 and 3 mm diameter) and calibrated with Crison standard buffer solutions at pH 4.01, 7.02, and 10.00, was used for pH measurements. The pH readings from the pH meter for D_2_O solutions are uncorrected for the effect of deuterium on glass electrodes [56]. Electrochemical measurements were carried out with a potentiostat CHI 1230B (CH Instruments, Inc., Bee Cave, TX, USA) in a standard three-electrode electrochemical cell. The working electrode was a glassy carbon (GC) electrode, the reference electrode was a KCl-saturated Ag/AgCl, and the counter was a Pt sheet. The GC electrode was carefully polished with alumina powder and then rinsed with distilled water and dried to obtain a reproducible surface for all the experiments. All the measurements, conducted in triplicate, were carried out on aqueous solutions containing the Pt(IV) compound at a concentration of 5 × 10^−^^4^ M (3 mg in 10 mL phosphate buffer, pH = 7.4, I = 0.1), and NaCl 0.5 M. A blank solution (without Pt(IV) complex) was also tested. All the peak potentials were measured with a scan rate of 20 mV s^−^^1^; the initial potential was chosen equal to the open-circuit potential, and the scan direction was negative. X-ray photoelectron spectroscopy (XPS) analyses were run on a PHI 5000 Versa Probe II Scanning XPS Microprobe spectrometer (ULVAC PHI Inc., Kanagawa, Japan). Measurements were carried out using a monochromatic Al Kα source (X-ray spot 200 µm) at a power of 50.8 W. Wide scan and detailed spectra (Pt4f, C1s, Cl2p, N1s, O1s) were acquired in Constant Analyzer Energy (CAE) mode with a pass energy of 117.40 and 29.35 eV, respectively. An electron gun was used for charge compensation (1.0 V, 20.0 µA). Data processing was performed by using the MultiPak software v. 9.9.0.8 (Ulvac-phi, Inc., Chigasaki, Japan).

### 3.2. Synthesis of cis,trans,cis-[PtCl_2_(OBz)_2_(cis-1,4-DACH)] (**1**; OBz = Benzoate = OOCC_6_H_5_)

Compound **1** was prepared following the procedure already reported in the literature [41].

### 3.3. Stability at Acidic pH

Compound **1** (0.5 mg, 0.0008 mmol) was dissolved in Acetone-d_6_ (550 µL) and treated with 100 µL of a solution of DCl (DCl in D_2_O, pH = 1.5). The mixture was kept at 37 °C and monitored by recording ^1^H-NMR spectra at different time intervals. A sample containing compound **1** in Acetone-d_6_/D_2_O (550/100 µL) was also prepared for comparison.

### 3.4. Reduction by Biological Reducing Agents

#### 3.4.1. Ascorbic Acid/Ascorbate

Compound **1** (0.5 mg, 0.0008 mmol) was dissolved in Acetone-d_6_ (550 µL) and placed into an NMR tube. A six-fold excess of ascorbic acid (0.85 mg, 0.005 mmol) and sodium ascorbate (0.95 mg, 0.005 mmol) dissolved in D_2_O (100 µL) were added into the NMR tube, and the resulting solution was monitored by recording ^1^H-NMR spectra at different time intervals (up to 5 days). After acquisition of the initial ^1^H-NMR spectrum, the sample was kept at 37 °C. Deconvolution of selected peaks was obtained by Origin (ver. 9.5; OriginLab Corporation, Northampton, MA, USA), and the corresponding areas were used to calculate the relative percentages of compound **1** and free benzoate at various time points.

#### 3.4.2. Glutathione

Compound **1** (0.5 mg, 0.0008 mmol) was dissolved in Acetone-d_6_ (550 µL) and placed into an NMR tube. A six-fold excess of glutathione (1.47 mg, 0.005 mmol) dissolved in D_2_O (100 µL) was added into the NMR tube, and the resulting solution was monitored by recording ^1^H-NMR spectra at different time intervals (up to 5 days). After acquisition of the initial ^1^H-NMR spectrum, the sample was kept at 37 °C. Deconvolution of selected peaks was obtained by Origin (ver. 9.5; OriginLab Corporation, Northampton, MA, USA), and the corresponding areas were used to calculate the relative percentages of compound **1** and free benzoate at various time points.

### 3.5. In Vivo Antitumor Activity

#### 3.5.1. Experiments with Animals

The mice were purchased from Charles River, Italy, housed in steel cages under controlled environmental conditions (constant temperature, humidity, and 12 h dark/light cycle), and alimented with commercial standard feed and tap water ad libitum.

#### 3.5.2. In Vivo Antitumor Activity in Lewis Lung Carcinoma (LLC)

The LLC cell line was purchased from ECACC, Salisbury, UK and maintained in DMEM (Euroclone, Pero, Italy) supplemented with 10% heat-inactivated fetal bovine serum (FBS; Euroclone), 10 mM *L*-glutamine, 100 U mL^−1^ penicillin, and 100 μg mL^−1^ streptomycin in a 5% CO_2_ air incubator at 37 °C. The LLC was implanted intramuscularly (*i.m.*) as a 2 × 10^6^ cell inoculum into the right hind leg of 8-week-old male and female C57BL mice (24 ± 3 g body weight). After 24 h from tumor implantation, mice were randomly divided into five groups (8 animals per group, 10 controls). Chemotherapy was delayed until the tumor became visible (day 7). From day 7 after tumor inoculation, the animals received daily vehicle solution (0.5% (*v*/*v*) of DMSO and 99.5% (*v*/*v*) of 0.9% NaCl water solution), compound **1** (5 mg kg^−1^ in vehicle solution) orally by gavage or cisplatin (1.5 mg kg^−1^ in 0.9% NaCl solution) intraperitoneally (*i.p.*). At day 15, the animals were sacrificed, their legs were amputated at the proximal end of the femur, and the inhibition of tumor growth was determined according to the difference in weight of the tumor-bearing leg and the healthy leg of the animals expressed as a percentage referring to the control animals. Body weight was measured every two days and was taken as a parameter for systemic toxicity. All the values are the means ± SD of not less than three measurements.

### 3.6. Neurotoxicity Studies

The dorsal root ganglia (DRG) from embryonic Sprague–Dawley rats (Envigo, San Pietro al Natisone, Italy) were aseptically removed and cultured onto a single layer of rat tail collagen surfaces in 35 mm dishes as previously described [54]. DRG were incubated in AN_2_ medium (Minimum Essential Medium plus 1.4 mM L-glutamine (Euroclone, Pero, Italy), 10% calf bovine serum (Hyclone, Thermo Scientific, Logan, UT, USA), 50 µg/mL ascorbic acid, 0.6% glucose (Sigma-Aldrich, St. Louis, MO, USA)), in the presence of 5 ng/mL nerve growth factor (NGF; Life Technologies, Monza, Italy) in 5% CO_2_ humidified incubator at 37 °C.

To evaluate the neurotoxicity of complex **1**, oxaliplatin (OXP) and kiteplatin, the DRG explants were treated for 2 h with NGF and subsequently exposed to each drug at different concentration for 24 or 48 h. OXP and kiteplatin were tested at 5, 7.5 and 10 µM, while complex **1** was tested at 0.5, 1.0, 2.5, and 5 µM concentrations. DRG treated with AN_2_ medium supplemented with 5 ng/mL NGF alone was used as controls. Phase-contrast micrographs were taken, and the length of the longest neurite in each DRG was measured by Image J (NIH, Bethesda, MD, USA), using a standard calibration grating photographed at the same magnification. A compound is considered neurotoxic when the mean neurite elongation is reduced by 50% or more after drug exposure vs. control.

## 4. Conclusions

The remarkably higher in vitro activity of complex **1** with respect to its Pt(II) precursor prompted us to extend the investigation to an in vivo system and to the possibility of oral administration. Therefore, the stability of complex **1** was investigated at acid pH (1.5) to mimic the stomach environment; the Pt(IV) prodrug was remarkably stable in acidic conditions and excess chloride ion undergoing a drop of the initial concentration to ~60% of the initial one only after 72 h at 37 °C. Moreover, the reduction of **1** by common bioreductants (ascorbic acid/sodium ascorbate and glutathione) was also rather slow (not complete after 120 h incubation at 37 °C), and as expected, it was faster with 2e^−^ reductant ascorbic acid/sodium ascorbate than with glutathione. The in vivo antitumor efficacy of compound **1** was then investigated in the LLC murine model by administering the drug orally and comparing the results with those obtained by treating the animals with the standard drug cisplatin administered intraperitoneally. A comparable reduction of tumor mass was observed in the two cases (~75%), thus indicating that there is the possibility of oral administration for this dibenzoato prodrug of kiteplatin. In vitro neurotoxicity studies indicated that compound **1** is neurotoxic at a concentration of ~0.5 µM for 48 h treatment, while it is cytotoxic at nM concentrations. An in vivo neurotoxicity test could better ascertain the potential of compound **1** as an antitumor drug suitable for oral administration.

## Figures and Tables

**Table 1:** *^a^* Vehicle (0.5% (*v*/*v*) of DMSO and 99.5% (*v*/*v*) of 0.9% NaCl in water). In vivo antitumor activity. Lewis lung carcinoma (LLC) was implanted *i.m.* (2 × 10^6^ cells inoculum) into the right hind leg of 8-week-old male and female C57BL mice (24 ± 3 g body weight). Chemotherapy was delayed until the tumor became visible (day 7). Days 7–14: animal received daily compound **1** (5 mg/kg in vehicle solution) orally by gavage or cisplatin (1.5 mg kg^−1^ in saline solution) *i.p.* At day 15, the animals were sacrificed, their legs were amputated at the proximal end of the femur, and the inhibition of tumor growth was determined as the difference in weight of the tumor-bearing leg and the healthy leg expressed as % referred to the control animals.

Compound	Daily Dose (mg/Kg)	Average Tumor Weight (Mean ± S.D., g)	Inhibition of Tumor Growth (%)
Control *^a^*	-	0.40 ± 0.07	-
1	5	0.11 ± 0.08	72.5
cisplatin	1.5	0.09 ± 0.02	77.5

## Data Availability

All data are available upon request from the Authors.
